# The Impact of Preoperative Malnutrition on Postoperative Outcomes in Older Patients Undergoing Lumbar Decompression

**DOI:** 10.7759/cureus.92060

**Published:** 2025-09-11

**Authors:** Yuki Taniguchi, Hideki Nakamoto, So Kato, Hiroyuki Nakarai, Kenichi Kono, Yuhei Saito, Reo Inoue, Hiroshi Okawa, Sakae Tanaka, Yasushi Oshima, Kazuhiko Fukatsu

**Affiliations:** 1 Surgical Center, The University of Tokyo Hospital, Tokyo, JPN; 2 Department of Orthopedic Surgery, The University of Tokyo Hospital, Tokyo, JPN

**Keywords:** decompression, geriatric, health-related quality of life, lumbar spine, malnutrition

## Abstract

Introduction: Malnutrition is closely linked to frailty or sarcopenia and is more likely to occur in elderly individuals. However, the impact of preoperative nutritional status on postoperative health-related quality of life following spine surgery remains unclear, even though the impact of nutritional status on the incidence of perioperative complications or mortality following spine surgery is well described. Therefore, this study aimed to investigate the impact of preoperative malnutrition on postoperative health-related quality of life in older patients following lumbar decompression.

Methods: We retrospectively analyzed our consecutive patients aged ≥ 65 years who underwent lumbar decompression. The primary outcome was assessed using the EuroQol five-dimension three-level questionnaire (EQ-5D-3L) administered preoperatively and one year postoperatively. Patients with preoperative geriatric nutritional risk index (GNRI) ≤ 98 were categorized as having malnutrition risk and were compared with those without such risk.

Results: We identified 245 eligible patients (144 males and 101 females), and 44 were categorized into the malnutrition risk group. The malnutrition risk group comprised a significantly lower proportion of patients with diabetes (4/44 [9.1%] vs 51/201 [25.4%], P = 0.02) and a higher proportion with rheumatoid arthritis (6/44 [13.6%] vs 6/201 [3.0%], P = 0.01). There were no significant between-group differences in one-year patient satisfaction (32/44 [72.7%] vs 152/201 [75.6%]; P = 0.70), preoperative EQ-5D-3L values (0.52 ± 0.17 vs 0.55 ± 0.16; P = 0.29), postoperative EQ-5D-3L values (0.66 ± 0.16 vs 0.70 ± 0.19; P = 0.17), or the improvement in EQ-5D-3L (0.14 ± 0.20 vs 0.16 ± 0.21; P = 0.69). However, the malnutrition risk group had more patients experiencing a deterioration in EQ-5D-3L at one year (10/44 [22.7%] vs 22/201 [11.0%], P = 0.047). Multivariate analysis revealed that preoperative GNRI ≤ 98 was an independent risk factor for a decrease in EQ-5D-3L values at one year (odds ratio, 2.64; 95% confidence interval, 1.08-6.20, P=0.03).

Conclusions: Despite comparable postoperative improvement in EQ-5D-3L between the two groups, a distinct subset of older patients with preoperative malnutrition risk exhibited a clinically significant deterioration in quality of life following lumbar decompression, underscoring the persistent vulnerability of this population.

## Introduction

The percentage of the world's population aged ≥65 has risen from 5.1% in 1950 to 8.2% in 2015 and is expected to rise to 17.8% by 2060, indicating a rapid aging of the world population over the next half-century [1]. With this aging population comes an associated increase in the prevalence of degenerative spinal disorders, leading to a higher demand for surgical treatment among older patients with these disabling conditions. However, the success of such surgical interventions may be influenced by various preoperative factors, among which nutritional status stands out as a crucial yet often overlooked determinant. Older adults are potentially at high risk of malnutrition, often accompanied by frailty or sarcopenia, and nutritional status is pivotal in overall health and healing processes, particularly in this population [2]. Despite the well-described impact of nutritional status on the incidence of perioperative complications or mortality following spine surgery, there is little information on its impact on the health-related quality of life (HRQOL) following spine surgery [3-7].

Therefore, this study aimed to investigate the impact of preoperative nutritional status on HRQOL, using the EuroQol five-dimension three-level (EQ-5D-3L) questionnaire at one year postoperatively in older patients who underwent lumbar decompression. Through this research, we aim to elucidate the complex relationship between preoperative nutritional status and surgical outcomes in this population. Finally, our findings aim to inform clinical practice, improve patient care pathways, and contribute to the ongoing efforts to improve the QOL and outcomes for older adults undergoing lumbar spine surgery.

## Materials and methods

Data source and patients

We performed a single-center retrospective cohort study including consecutive patients aged ≥65 years who underwent elective lumbar decompression at our institution between April 1, 2017, and July 31, 2022. Exclusion criteria included revision surgery, procedures involving instrumentation or fusion, and decompression extending into the thoracic spine. Patients with unavailable preoperative or postoperative patient-reported outcome measures (PROMs) were also excluded. Written informed consent was obtained from all participants.

Data collection

The collected data included patient characteristics, surgery-related data, and PROMs. Patient characteristics included age, sex, body mass index (BMI), diagnosis, preoperative neurological symptom (cauda equina syndrome or radiculopathy), preoperative American Society of Anesthesiologists Physical Status (ASA-PS), smoking status, regular use of steroids, and comorbidities, including diabetes mellitus, rheumatoid arthritis, and hemodialysis. Patients’ ages were categorized into three groups: 65-74, 75-84, and 85+.

Surgery-related data included operation time, amount of blood loss, surgical procedure (laminectomy only or laminectomy with discectomy), number of decompressed levels, and the use of microendoscopy. Incidental durotomy that occurred intraoperatively was also recorded.

In this study, HRQOL was assessed using the EQ-5D-3L instrument preoperatively and at one year postoperatively [8]. For the subsequent analysis, EQ-5D health states were converted into a single summary number [9]. The EuroQOL International Group provided permission to use the instrument via email. The questionnaire was already available in Japanese on the EuroQOL website (https://euroqol.org/wp-content/uploads/2023/11/EQ-5D-3LUserguide-Japanese-23-07.pdf). Additionally, postoperative patient satisfaction was evaluated one year postoperatively using a seven-point Likert scale (very satisfied, satisfied, slightly satisfied, neither satisfied nor dissatisfied, slightly dissatisfied, dissatisfied, and very dissatisfied) [10]. Patients indicating being very satisfied, satisfied, or slightly satisfied were classified as satisfied, whereas those reporting any other response were categorized as dissatisfied.

Assessment of preoperative nutritional status

Preoperative nutritional status was evaluated using the geriatric nutritional risk index (GNRI), calculated from the patient's preoperative serum albumin level and BMI [11]. Patients with a preoperative GNRI ≤ 98 were classified as being at malnutrition risk, as previously described [12,13].

Statistical analysis

Continuous variables were compared using the t-test. Fisher’s exact test was used for comparisons of 2×2 categorical data, while the chi-square test was applied for other categorical data. Wilcoxon signed-rank test was used for the nonparametric paired data analysis. The t-value for the t-test, the chi-square value for the chi-square test, and the S statistic for the Wilcoxon signed-rank test were reported, respectively. Multivariate logistic regression analysis was conducted to determine risk factors associated with decreased EQ-5D-3L values at one year postoperatively compared with the preoperative levels. Explanatory variables for the multivariate analysis were selected based on those with P-values < 0.1 in the univariate analysis, in addition to basic demographic factors, including sex and age. Statistical significance was set at P < 0.05. All statistical analyses were performed using JMP Pro 17 software (SAS Institute Inc., Cary, NC, USA).

## Results

Patient characteristics

We identified 245 eligible patients in our cohort, comprising 144 males and 101 females. Table 1 summarizes the detailed data of the study population. The mean age at surgery was 75.8 ± 6.0 with over half of the patients being categorized into the 75-84 years age group (Table 1). The mean preoperative GNRI was 106.1 ± 8.3, indicating a generally good preoperative nutritional status among the study population (Table 1). Diabetes was present in 55 patients (22.4%), and most (75.9%) were categorized in ASA-PS 2 preoperatively (Table 1). Laminectomy was performed on 215 patients (87.8%), whereas the remaining 30 patients (12.2%) underwent laminectomy with discectomy, and 160 patients (65.3%) underwent microendoscopic surgery (Table 1). Surgery was conducted for cauda equina syndrome in 175 patients (71.4%) and for radiculopathy in the remaining 70 patients (28.6%) (Table 1). Incidental durotomy was identified in 25 patients (10.2%) (Table 1). The mean EQ-5D-3L value of the whole study population significantly improved from 0.54 to 0.70 (P < 0.0001, S statistic=11399.5) (Table 1).

**Table 1:** Patient characteristics, surgery-related data, and the EQ-5D-3L results of the study population Values are presented as n (%) or mean [SD]. SD, standard deviation; GNRI, geriatric nutritional risk index; ASA-PS, American Society of Anesthesiologists Physical Status; EQ-5D-3L, EuroQol five-dimension three-level questionnaire [8]

Number of patients	245	(%)
Sex		
Male	144	(58.8)
Female	101	(41.2)
Age at surgery		
65-74	99	(40.4)
75-84	129	(52.7)
85+	17	(6.9)
Body mass index	24.2	[3.7]
Preoperative GNRI	106.1	[8.3]
ASA-PS		
1	29	(11.8)
2	186	(75.9)
3	30	(12.2)
Smoking	12	(4.9)
Comorbidities		
Diabetes	55	(22.4)
Rheumatoid arthritis	12	(4.9)
Hemodialysis	1	(0.4)
Regular use of steroids	23	(9.4)
Operation time, min	125.7	[46.1]
Blood loss, ml	79.1	[100.8]
Surgical procedure		
Laminectomy	215	(87.8)
Laminectomy with discectomy	30	(12.2)
Neurological symptom		
Cauda equina syndrome	175	(71.4)
Radiculopathy	70	(28.6)
Number of levels decompressed	1.5	[0.8]
Microendoscopic surgery	160	(65.3)
Incidental durotomy	25	(10.2)
EQ-5D-3L		
Preoperative	0.54	[0.16]
One-year postoperatively	0.70	[0.18]

Comparison between malnutrition risk and no-risk groups

The malnutrition risk group with preoperative GNRI ≤ 98 included 44 patients, whereas the remaining 201 patients with preoperative GNRI > 98 were categorized into the no-risk group (Table 2). Regarding patient characteristics, patients in the malnutrition risk group had a significantly lower BMI as expected (Table 2). The malnutrition risk group included a significantly lower proportion of patients with diabetes and a higher proportion of those with rheumatoid arthritis (Table 2). The two groups had no significant differences in patient satisfaction at one year, preoperative and postoperative EQ-5D-3L values, and the increase in EQ-5D-3L values (Table 2). EQ-5D-3L values significantly improved in both groups (P < 0.0001 for both groups). There was no significant difference between the two groups in the proportion of patients who achieved a 22.0% improvement in EQ-5D-3L, previously reported as the minimal clinically important difference (MCID) in EQ-5D-3L values for patients undergoing lumbar decompression (Table 2) [14]. However, the malnutrition risk group included a significantly higher proportion of the patients who experienced EQ-5D-3L deterioration at one year (22.7% vs 11.0%, P=0.047) (Table 2).

**Table 2:** Comparison between the malnutrition risk and no-risk groups Values are presented as n (%) or mean [SD]. Statistical significance was set at P* < 0.05. Continuous variables were compared using the t-test. Fisher’s exact test was used for comparisons of 2×2 categorical data, while the chi-square test was applied for other categorical data. The t-values for the t-test and the chi-square values for the chi-square test were reported, respectively. GNRI, geriatric nutritional risk index; SD, standard deviation; ASA-PS, American Society of Anesthesiologists Physical Status; EQ-5D-3L, EuroQol five-dimension three-level questionnaire [8]

	Malnutrition risk group	No-risk group	P	t-value	Chi-square value
Number of patients	44 (18.0)	201 (82.0)			
Sex			0.13		
Male	21 (47.4)	123 (61.2)			
Female	23 (52.3)	78 (38.8)			
Age at surgery			0.82		0.40
65–74	16 (36.4)	83 (41.3)			
75–84	25 (56.8)	104 (51.7)			
85–	3 (6.8)	14 (7.0)			
Body mass index	20.1 [2.4]	25.1 [3.3]	<0.001*	-9.7	
Preoperative GNRI	94.3 [4.1]	108.7 [6.5]	<0.001*	-14.0	
ASA-PS			0.31		2.33
1	8 (18.2)	21 (10.5)			
2	32 (72.7)	154 (76.6)			
3	4 (9.1)	26 (12.9)			
Smoking	1 (2.3)	11 (5.5)	0.70		
Comorbidities					
Diabetes	4 (9.1)	51 (25.4)	0.02*		
Rheumatoid arthritis	6 (13.6)	6 (3.0)	0.01*		
Hemodialysis	1 (2.3)	0 (0.0)	0.18		
Regular use of steroids	7 (15.9)	16 (8.0)	0.15		
Operation time, min	117.3 [42.9]	127.6 [46.7]	0.18	-1.34	
Blood loss, ml	61.2 [73.0]	83.0 [105.7]	0.19	-1.30	
Surgical procedure			0.80		
Laminectomy	38 (86.4)	177 (88.1)			
Laminectomy with discectomy	6 (13.6)	24 (11.9)			
Neurological symptom			0.27		
Cauda equina syndrome	28 (63.6)	147 (73.1)			
Radiculopathy	16 (36.4)	54 (26.9)			
Number of levels decompressed	1.4 [0.8]	1.6 [0.9]	0.25	-1.16	
Microendoscopic surgery	31 (70.5)	129 (64.2)	0.49		
Incidental durotomy	1 (2.3)	24 (11.9)	0.06		
Patients’ satisfaction			0.70		
Satisfied	32 (72.7)	152 (75.6)			
Dissatisfied	12 (27.3)	49 (24.4)			
EQ-5D-3L value					
Preoperative	0.52 [0.17]	0.55 [0.16]	0.29	-1.06	
One-year postoperatively	0.66 [0.16]	0.70 [0.19]	0.17	-1.38	
Amount of change	0.14 [0.20]	0.16 [0.21]	0.69	-0.40	
Number of patients with a percentage change in EQ-5D-3L ≥ 22.0	22 (50.0)	99 (49.3)	1.00		
Number of patients with the one-year change in EQ-5D-3L < 0	10 (22.7)	22 (11.0)	0.047*		

Risk factors for EQ-5D-3L deterioration

To determine the actual impact of preoperative nutritional status on postoperative EQ-5D-3L deterioration, we conducted univariate and multivariate analyses. In the univariate analysis, EQ-5D-3L deterioration was significantly associated with preoperative GNRI ≤ 98 (P = 0.048), while its association with ASA-PS classification was marginal (P = 0.06) (Table 3). Multivariate logistic regression analysis identified preoperative GNRI ≤ 98 as an independent risk factor for deterioration in EQ-5D-3L values at one year postoperatively (odds ratio, 2.64; 95% confidence interval, 1.08-6.20, P=0.03) (Table 3).

**Table 3:** Univariate and multivariate logistic regression analyses for EQ-5D-3L deterioration at one year postoperatively Statistical significance was set at P* < 0.05. EQ-5D-3L, EuroQol five-dimension three-level questionnaire [8]; GNRI, geriatric nutritional risk index; ASA-PS, American Society of Anesthesiologists Physical Status; CI, confidence interval

	Univariate analysis		Multivariate analysis	
	Odds ratio [CI]	P	Odds ratio [CI]	P
Age, years		0.45		0.45
85–	2.04 [0.51–6.83]		1.73 [0.42–6.11]	
75–84	0.87 [0.39–1.95]		0.76 [0.33–1.74]	
65–74	Reference		Reference	
Sex		0.94		0.57
Male	1.03 [0.49–2.24]		1.25 [0.57–2.82]	
Female	Reference		Reference	
Preoperative GNRI		0.048*		0.03*
≤ 98	2.39 [1.01–5.40]		2.64 [1.08–6.20]	
> 98	Reference		Reference	
ASA-PS		0.06		0.051
3	0.47 [0.02–5.13]		0.50 [0.02–5.68]	
2	2.49 [0.69–15.98]		2.82 [0.75–18.52]	
1	Reference		Reference	
Smoking		0.25		
Yes	2.34 [0.50–8.39]			
No	Reference			
Diabetes		0.22		
Yes	1.70 [0.72–3.76]			
No	Reference			
Rheumatoid arthritis		0.25		
Yes	2.34 [0.50–8.39]			
No	Reference			
Hemodialysis		0.60		
Yes	<0.0001 [**–39.2]			
No	Reference			
Regular use of steroids		1.00		
Yes	1.00 [0.23–3.15]			
No	Reference			
Operation time, min		0.13		
≥ 120	0.56 [0.25–1.19]			
< 120	Reference			
Blood loss, ml		0.74		
≥ 100	0.87 [0.36–1.93]			
< 100	Reference			
Surgical procedure		0.58		
Laminectomy	1.40 [0.46–6.13]			
Laminectomy with discectomy	Reference			
Neurological symptom		0.95		
Cauda equina syndrome	1.03 [0.46–2.45]			
Radiculopathy	Reference			
Number of levels decompressed		0.17		
≥ 2	1.69 [0.79–3.59]			
1	Reference			
Microendoscopic surgery		0.72		
Yes	0.87 [0.41–1.92]			
No	Reference			
Incidental durotomy		0.40		
Yes	0.55 [0.09–2.00]			
No	Reference			

## Discussion

This study revealed two main findings. First, the preoperative nutritional status has minimal impact on the one-year postoperative HRQOL in older patients undergoing lumbar decompression. Second, despite the comparable postoperative improvement in HRQOL between the two groups, some patients with preoperative malnutrition risk exhibited HRQOL deterioration at one year compared with those without the risk.

Previous studies have examined the relationship between preoperative nutritional status and perioperative complications following lumbar spine surgery. Puvanesarajah et al. found that malnutrition significantly increased surgical site infection, one-year mortality rates, and length of stay in older patients undergoing lumbar spine fusion [3]. Kang et al. identified a significant association between preoperative serum albumin level and postoperative complications in patients undergoing fusion over three or more levels for degenerative lumbar spinal disease [4]. Elsamadicy et al. revealed that malnourished patients defined based on preoperative serum albumin levels had significantly higher rates of perioperative adverse events, unplanned readmission, and prolonged length of stay than nourished patients following lumbar fusion for spondylolisthesis [5]. However, no reports have investigated the association between preoperative nutritional status and postoperative HRQOL following such surgeries. Malnutrition has been closely linked to sarcopenia and frailty, and recent reports have emerged regarding the association between sarcopenia/frailty and postoperative HRQOL following lumbar spine surgery [15-17]. Concerning sarcopenia, some reports suggest no correlation with postoperative PROMs, whereas conflicting reports exist regarding the correlation between preoperative frailty and postoperative PROMs [18,19]. In this study, preoperative malnutrition had no association with HRQOL or postoperative satisfaction one year postoperatively. This discrepancy, despite the close relationship between frailty and malnutrition, might be because most of the 44 cases with malnutrition risk in this cohort were those at mild nutrition-related risk (92 ≤ GNRI ≤ 98), with only nine cases being at moderate (82 ≤ GNRI < 92) or severe nutrition-related risk (GNRI < 82) [11]. Therefore, in this study, we might have underestimated the impact of malnutrition on postoperative HRQOL.

Conversely, our study identified preoperative malnutrition as an independent risk factor for the incidence of decreased EQ-5D-3L values one year postoperatively. However, the clinical significance of the decline in EQ-5D-3L from baseline is unclear. There have been a few reports on the MCID in improving EQ-5D-3L following lumbar spine surgery; however, no report elucidates the MCID in the EQ-5D-3L deterioration [14,20,21]. This is unsurprising since MCIDs are usually estimated as improvement rather than deterioration thresholds. EQ-5D is expected to improve following lumbar spine surgery, and a previous study demonstrated that EQ-5D scores have shown improvement from 0.31 preoperatively to 0.41 at one year postoperatively, even in patients reporting their treatment outcomes as unsuccessful after lumbar spine surgery [22]. Therefore, it might be appropriate to interpret EQ-5D deterioration at one year postoperatively as a significant change. Given the close relationship between malnutrition and sarcopenia/frailty, it is plausible to assume that some older patients at preoperative malnutrition risk might have failed to resume their activities of daily living, possibly owing to decreased physical performance caused by impaired muscle strength. Further investigation is warranted to determine the MCIDs in EQ-5D-3L deterioration following lumbar spine surgery. Additionally, the next challenge is determining whether preoperative nutritional therapy, such as administering oral nutrition supplements and nutritional counseling by dietitians, can improve the clinical course of patients experiencing deterioration in postoperative HRQOL following lumbar surgery.

In this cohort, the malnutrition risk group included a significantly lower proportion of patients with diabetes and a higher proportion of those with rheumatoid arthritis. Increased BMI correlates with a higher incidence of diabetes, and BMI is used to calculate GNRI; therefore, it is understandable that the incidence of diabetes was higher in patients with a high GNRI preoperatively [23]. Regarding rheumatoid arthritis, it is reported that patients with this condition have a high risk of malnutrition, particularly among those aged ≥65, with approximately one-third reported to have impaired nutritional status [24,25]. Therefore, our study results were considered to be consistent with these previous findings.

Additionally, there is room for discussion regarding selecting indicators for preoperative nutritional status. Notably, numerous indices have been proposed to assess nutritional status in literature, including GNRI, prognostic nutritional index (PNI), mini nutritional assessment-short form (MNA-SF), controlling nutritional status (CONUT) score, global leadership initiative on malnutrition (GLIM) criteria, malnutrition universal screening tool (MUST), and others [11,26-30]. In a study by Acarbaş et al., GNRI outperformed the PNI and CONUT scores in predicting perioperative adverse medical events among older patients undergoing spinal surgery [31]. Their study suggested that GNRI might accurately reflect patients' nutritional status, affecting the outcome of spine surgery in older adults. Consequently, we used the GNRI in our study. However, further research is needed to determine which nutritional index is most closely associated with postoperative HRQOL in patients undergoing lumbar spine surgery.

This study has some limitations. First, the data were collected retrospectively and not prospectively; therefore, information bias might have occurred. Second, because data on comorbidities that could markedly affect QOL (e.g., stroke and other cerebrovascular/brain diseases, dementia, cardiopulmonary diseases, orthopedic conditions, and malignancies) were unavailable, the possibility of omitted variable bias cannot be ruled out. Third, some selection bias may exist regarding patients who underwent surgery. Notably, some patients might have been unable to undergo surgery due to underlying diseases impacting their nutritional status.

## Conclusions

We investigated the effect of preoperative nutritional status on HRQOL at one year postoperatively in older patients undergoing lumbar decompression. Preoperative nutritional status has little impact on the postoperative one-year HRQOL in these patients. However, despite the comparable postoperative improvement in EQ-5D-3L between the preoperative malnutrition risk group and the no-risk group, some patients at preoperative malnutrition risk showed HRQOL deterioration at one year postoperatively. Although malnutrition risk, defined as a preoperative GNRI ≤ 98, was identified as an independent risk factor for decreased HRQOL after lumbar decompression in older adults, whether preoperative nutritional intervention can prevent such deterioration in HRQOL remains an issue for future investigation.

## References

[1] (2019). World population prospects 2019: highlights. https://population.un.org/wpp/assets/Files/WPP2019_Highlights.pdf.

[2] Kaiser MJ, Bauer JM, Rämsch C (2010). Frequency of malnutrition in older adults: a multinational perspective using the mini nutritional assessment. J Am Geriatr Soc.

[3] Puvanesarajah V, Jain A, Kebaish K (2017). Poor nutrition status and lumbar spine fusion surgery in the elderly: readmissions, complications, and mortality. Spine (Phila Pa 1976).

[4] Kang T, Park SY, Lee JS, Lee SH, Park JH, Suh SW (2020). Predicting postoperative complications in patients undergoing lumbar spinal fusion by using the modified five-item frailty index and nutritional status. Bone Joint J.

[5] Elsamadicy AA, Havlik J, Reeves BC (2021). Effects of preoperative nutritional status on complications and readmissions after posterior lumbar decompression and fusion for spondylolisthesis: a propensity-score analysis. Clin Neurol Neurosurg.

[6] Ushirozako H, Hasegawa T, Yamato Y (2021). Does preoperative prognostic nutrition index predict surgical site infection after spine surgery?. Eur Spine J.

[7] Lee NJ, Kothari P, Kim JS, Phan K, Di Capua J, Shin J, Cho SK (2017). Nutritional status as an adjunct risk factor for early postoperative complications following posterior cervical fusion. Spine (Phila Pa 1976).

[8] EuroQol Group (1990). EuroQol--a new facility for the measurement of health-related quality of life. Health Policy.

[9] Tsuchiya A, Ikeda S, Ikegami N (2002). Estimating an EQ-5D population value set: the case of Japan. Health Econ.

[10] Nakajima K, Miyahara J, Ohtomo N (2023). Impact of body mass index on outcomes after lumbar spine surgery. Sci Rep.

[11] Bouillanne O, Morineau G, Dupont C (2005). Geriatric Nutritional Risk Index: a new index for evaluating at-risk elderly medical patients. Am J Clin Nutr.

[12] Sasaki M, Miyoshi N, Fujino S (2020). The Geriatric Nutritional Risk Index predicts postoperative complications and prognosis in elderly patients with colorectal cancer after curative surgery. Sci Rep.

[13] Sargento L, Vicente Simões A, Rodrigues J, Longo S, Lousada N, Palma Dos Reis R (2017). Geriatric nutritional risk index as a nutritional and survival risk assessment tool in stable outpatients with systolic heart failure. Nutr Metab Cardiovasc Dis.

[14] Nakarai H, Kato S, Kawamura N (2022). Minimal clinically important difference in patients who underwent decompression alone for lumbar degenerative disease. Spine J.

[15] Crovetto Mattassi M, Henríquez Mella C, Pérez Bocaz L (2022). Association between sarcopenia and nutritional status in Chilean older people aged 65 years and older. Nutrients.

[16] Papadopoulou SK, Papadimitriou K, Voulgaridou G, Georgaki E, Tsotidou E, Zantidou O, Papandreou D (2021). Exercise and nutrition impact on osteoporosis and sarcopenia-the incidence of osteosarcopenia: a narrative review. Nutrients.

[17] Eglseer D, Eminovic S, Lohrmann C (2016). Association between sarcopenia and nutritional status in older adults: a systematic literature review. J Gerontol Nurs.

[18] Chotai S, Gupta R, Pennings JS (2022). Frailty and sarcopenia: impact on outcomes following elective degenerative lumbar spine surgery. Spine (Phila Pa 1976).

[19] Beauchamp-Chalifour P, Flexman AM, Street JT (2021). The impact of frailty on patient-reported outcomes after elective thoracolumbar degenerative spine surgery. J Neurosurg Spine.

[20] Solberg T, Johnsen LG, Nygaard ØP, Grotle M (2013). Can we define success criteria for lumbar disc surgery? : estimates for a substantial amount of improvement in core outcome measures. Acta Orthop.

[21] Burgstaller JM, Wertli MM, Ulrich NH (2020). Evaluating the minimal clinically important difference of EQ-5D-3L in patients with degenerative lumbar spinal stenosis: a Swiss prospective multicenter cohort study. Spine (Phila Pa 1976).

[22] Hansson-Hedblom A, Jonsson E, Fritzell P, Hägg O, Borgström F (2019). The association between patient reported outcomes of spinal surgery and societal costs: a register based study. Spine (Phila Pa 1976).

[23] Sattar N, Gill JM (2014). Type 2 diabetes as a disease of ectopic fat?. BMC Med.

[24] Doubek JG, Kahlow BS, Nisihara R, Skare TL (2022). Rheumatoid arthritis and nutritional profile: a study in Brazilian females. Int J Rheum Dis.

[25] Cano-García L, Redondo-Rodríguez R, Manrique-Arija S (2023). Prevalence of malnutrition and associated factors in older patients with rheumatoid arthritis: a cross-sectional study. Nutrients.

[26] Onodera T, Goseki N, Kosaki G (1984). [Prognostic nutritional index in gastrointestinal surgery of malnourished cancer patients]. Nihon Geka Gakkai Zasshi.

[27] Kondrup J, Allison SP, Elia M (2003). ESPEN guidelines for nutrition screening 2002. Clin Nutr.

[28] Ignacio de Ulíbarri J, González-Madroño A, de Villar NG (2005). CONUT: a tool for controlling nutritional status. First validation in a hospital population. Nutr Hosp.

[29] Jensen GL, Cederholm T, Correia MI (2019). GLIM criteria for the diagnosis of malnutrition: a consensus report from the global clinical nutrition community. JPEN J Parenter Enteral Nutr.

[30] Stratton RJ, Hackston A, Longmore D (2004). Malnutrition in hospital outpatients and inpatients: prevalence, concurrent validity and ease of use of the 'malnutrition universal screening tool' ('MUST') for adults. Br J Nutr.

[31] Acarbaş A, Baş NS (2021). Which objective nutritional index is better for the prediction of adverse medical events in elderly patients undergoing spinal surgery?. World Neurosurg.

